# Prevalence of fibromyalgia in a Brazilian series of patients with multiple sclerosis

**DOI:** 10.1055/s-0043-1772673

**Published:** 2023-10-04

**Authors:** Cinthia Thomas, Bianca Thais Schneider, Caroline Schiochet Verza, Gabriel Fassina, Laís Restel Weber, Marlinton Moreira, Paula Tormen Fusinato, Cassiano Mateus Forcelini

**Affiliations:** 1 Hospital São Vicente de Paulo, Passo Fundo RS, Brazil.; 2 Universidade de Passo Fundo, Faculdade de Medicina, Passo Fundo RS, Brazil.

**Keywords:** Multiple Sclerosis, Pain, Fibromyalgia, Comorbidity, Depression, Anxiety, Esclerose Múltipla, Dor, Fibromialgia;, Comorbidade, Depressão, Ansiedade

## Abstract

**Background** The prevalence of pain in patients with multiple sclerosis is remarkable. Fibromyalgia has been considered as one of the forms of chronic pain encompassed in multiple sclerosis, but data are restricted to studies from Europe and North America.

**Objective** To assess the prevalence of fibromyalgia in a series of Brazilian patients with multiple sclerosis and the characteristics of this comorbidity.

**Methods** The present cross-sectional study included 60 consecutive adult patients with multiple sclerosis. Upon consent, participants underwent a thorough evaluation for disability, fatigue, quality of life, presence of fibromyalgia, depression, and anxiety.

**Results** The prevalence of fibromyalgia was 11.7%, a figure similar to that observed in previous studies. Patients with the comorbidity exhibited worse scores on fatigue (median and interquartile range [IQR]: 68 [48–70] versus 39 [16.5–49]; *p* < 0.001), quality of life (mean ± standard deviation [SD]: 96.5 ± 35.9 versus 124.8 ± 28.8; *p* = 0.021), anxiety (mean ± SD: 22.7 ± 15.1 versus 13.8 ± 8.4; *p* = 0.021), and depression (median and IQR: 23 [6–28] versus 6 [3–12.5]; *p* = 0.034) indices than patients without fibromyalgia. There was a strong positive correlation between depression and anxiety scores with fatigue (r = 0.773 and r = 0.773, respectively; p < 0.001). Conversely, a moderate negative correlation appeared between the Expanded Disability Status Scale (EDSS), fatigue, and depression scores with quality of life (r= −0.587, r= −0.551, r= −0.502, respectively; *p* < 0.001).

**Conclusion** Fibromyalgia is a comorbidity of multiple sclerosis that can enhance fatigue and decrease quality of life, although depression, anxiety, and disability are factors that can potentiate the impact of the comorbidity.

## Resumo

**Antecedentes** A prevalência de dor em pacientes com esclerose múltipla é significativa. A fibromialgia é considerada uma forma de dor crônica encontrada na esclerose múltipla, mas os dados são restritos a estudos europeus e da América do Norte.

**Objetivo** Avaliar a prevalência de fibromialgia em uma série de pacientes com esclerose múltipla e as características desta comorbidade.

**Métodos** O presente estudo transversal incluiu consecutivamente 60 pacientes adultos com esclerose múltipla. Após o consentimento, os participantes foram submetidos à avaliação para determinação de incapacidade, fadiga, qualidade de vida, presença de fibromialgia, depressão e ansiedade.

**Resultados** A prevalência de fibromialgia foi de 11,7%, similar ao observado em estudos prévios. Pacientes com a comorbidade apresentaram piores escores de fadiga (mediana e intervalo interquartil [IIQ]: 68 [48–70] *versus* 39 [16,5–49]; *p* < 0,001], qualidade de vida (média ± desvio padrão [DP]: 96,5 ± 35,9 *versus* 124,8 ± 28,8; *p* = 0,021), ansiedade (média ± DP: 22,7 ± 15,1 *versus* 13,8 ± 8,4; *p* = 0,021) e depressão (mediana e IIQ: 23 (6–28) *versus* 6 (3–12,5); *p* = 0,034] do que pacientes sem fibromialgia. Houve correlação positiva forte dos escores de depressão e de ansiedade com a fadiga (r = 0,773 e r = 0,773, respectivamente; *p* < 0,001). Concomitantemente, houve correlação negativa moderada entre os escores de escala de estado de incapacidade expandida, fadiga e depressão com a qualidade de vida (r = - 0,587, r = - 0,551, r = - 0,502, respectivamente; *p* < 0,001).

**Conclusão** A fibromialgia é uma comorbidade de esclerose múltipla que pode aumentar a fadiga e diminuir a qualidade de vida, embora depressão, ansiedade e incapacidade sejam fatores potencializadores dessa morbidade associada.

## INTRODUCTION

Multiple sclerosis is a common chronic demyelinating disease of the central nervous system (CNS) with rising figures of global prevalence in the last decade. 1 This condition places a huge economic burden on healthcare systems and societies in low- and middle-income countries, 2 just where the frequency of its comorbidities is less known. 3

The prevalence of pain in adult patients with multiple sclerosis is of ∼ 63%, with diversified characteristics, not only neuropathic. 4 This enables the possibility of concomitant occurrence of other common painful conditions as fibromyalgia, which has been recently considered as one of the forms of chronic pain encompassed in multiple sclerosis. Prevalence of fibromyalgia in adult patients with multiple sclerosis was reported as 17.3 and 19.4% in single centers located in Italy and Turkey, respectively. 5 6 A regional survey performed in Manitoba, Canada, found a prevalence of fibromyalgia diagnosis of 6.82% in multiple sclerosis patients, but of 3.04% in the general population. 7

In Brazil, the prevalence rate of multiple sclerosis reaches up to 27.2/100,000 inhabitants. 8 On the other hand, ∼ 2% of the Brazilian population is affected by fibromyalgia. 9 However, the frequency of fibromyalgia in Brazilian patients with multiple sclerosis and the characteristics of this comorbidity are unknown. This is the reason why we attempted to explore this issue.

## METHODS

The present cross-sectional study was conducted in the Hospital São Vicente de Paulo (HSVP), in Passo Fundo – RS, Brazil. All adult patients with multiple sclerosis consecutively assisted by the neurological staff (Instituto de Neurologia e Neurocirurgia [INN]) from August 2021 to December 2022 were invited to participate in the study. The present survey was approved by the local ethics committee (approval number 4.737.086, from May 26th, 2021). Only one patient declined participation.

Upon written consent, the participants underwent a thorough evaluation for disability, fatigue, quality of life, presence of fibromyalgia, depression, and anxiety. Disability was measured with the Expanded Disability Status Scale (EDSS; the higher the score, the greater the disability). 10 The assessment of fatigue was performed using the Modified Fatigue Impact Scale (the higher the score, the greater the degree of fatigue). 11 The Functional Assessment of Multiple Sclerosis quality of life instrument (FAMS) was employed for evaluating quality of life (the higher the score, the better the quality of life). 12 Fibromyalgia was diagnosed according to the American College of Rheumatology modified criteria: 1. a score in the Widespread Pain Index (WPI) ≥ 7 and a score ≥ 5 in the Symptom Severity (SS) scale, or a score in the WPI from 3 to 6 and a score in the SS scale ≥ 9; 2. presence of symptoms at a similar level for at least 3 months; 3. absence of a disorder that would otherwise explain the pain. 13 The presence of depression and anxiety as comorbidities were also assessed with the aid of the Beck Depression Inventory (BDI) and Hamilton Anxiety Rating Scale (HARS), respectively. 14 15 A score > 9 in the BDI was used for defining the presence of depression, and > 11 in the HARS for anxiety. Demographic and clinical data was obtained from history and medical records. Patients with relapse of multiple sclerosis were evaluated only after stabilization.

The Fisher exact test was employed for the analysis of qualitative variables. The Student *t* test was used for comparing quantitative data, while the corresponding tool in case of asymmetric distribution was the Mann-Whitney U test. Correlation between quantitative data was accomplished with the Pearson correlation coefficient. The *p* -value for significance was established as 0.05.

## RESULTS

Our original sample was composed by 61 multiple sclerosis patients, but due to a refusal the sample resulted in 60 participants, whose demographic and clinical characteristics are depicted in Table 1 . Most were Caucasian, reflecting the local ethnic composition. As expected, women comprised the majority of the population included in the study.

**Table 1:** Clinical and demographic characteristics of the sample ( *n* = 60).

Characteristics		Results
Female		75%
Caucasian		86.7%
Age (years old; mean ± SD)		40.4 ± 12.3
Body mass index (kg/m ^2^ ; mean ± SD)		26.2 ± 4.8
Duration of disease (years; mean ± SD)		12.6 ± 8.7
Time since diagnosis (years; mean ± SD)		9.9 ± 7.1
Time since the first treatment (years; median and IQR)		8 (3.2–14)
Progressive forms (primary and secondary)		16.7%
Smoking		13.3%
Alcohol intake		35%
Presence of fibromyalgia		11.7%
Treatment for multiple sclerosis	On current treatment	95%
	Changed treatment	41.7%
EDSS (median and IQR)		2 (1–3.5)
Presence of depression		41.7%
Presence of anxiety		58.3%

Abbreviation: EDSS, Expanded Disability Status Scale; IQR, interquartile range; SD, standard deviation.

Notes: Qualitative variables are presented as percentage, while quantitative data is expressed as mean ± SD or median and IQR, according to distribution (normal or asymmetric).

Patients with and without fibromyalgia were compared and the results are presented in Table 2 . Only four variables exhibited significant differences between the groups: fatigue index, quality of life index, anxiety score, and depression score.

**Table 2:** Comparison of clinical characteristics between patients with and without fibromyalgia.

Characteristics	Fibromyalgia ( *n* = 7)	Non-fibromyalgia ( *n* = 53)	*p-value*
Female	6	39	0.668
Caucasian	5	47	0.232
Smoking	1	7	1.000
Alcohol intake	2	19	1.000
Progressive forms of multiple sclerosis	2	8	0.330
Change of treatment for multiple sclerosis	1	24	0.127
Age (years old)	41 ± 11.7	40.3 ± 12.5	0.892
Body mass index (kg/m ^2^ )	27.6 (21–33.6)	25.5 (23.5–27.6)	0.705
Duration of disease (years)	12.2 ± 11.3	12.7 ± 8.5	0.896
Time since diagnosis (years)	6.5 ± 5.1	10.4 ± 7.2	0.180
Time since the first treatment (years)	7.5 (1–10)	8 (3–14)	0.250
EDSS	3 (2.5–5.5)	2 (1–3.5)	0.065
Fatigue index	68 (48–70)	39 (16.5–49)	< 0.001*
Quality of life index	96.5 ± 35.9	124.8 ± 28.8	0.021*
Anxiety score	22.7 ± 15.1	13.8 ± 8.4	0.021*
Depression score	23 (6–28)	6 (3–12.5)	0.034*
Presence of anxiety	5	30	0.688
Presence of depression	5	20	0.117

Abbreviation: EDSS, Expanded Disability Status Scale.

Notes: Percentages were compared with the Fisher exact test (two-sided); quantitative variables were compared by the Student *t* test or the Mann-Whitney U test, according to distribution in each group (normal or asymmetric); *significant difference.

Qualitative variables are presented as absolute count, while quantitative data is expressed as mean ± SD or median and IQR, according to distribution.

We performed the correlations between main quantitative data with special interest on fatigue index and quality of life index. These results are presented in Table 3 .

**Table 3:** Correlation of quantitative variables with fatigue index and quality of life index ( *n* = 60).

Correlation		r †	*p-value*	Interpretation of correlation
**Fatigue index**	Age	0.090	0.496	Very weak
	Body mass index	0.051	0.697	Very weak
	Duration of the disease	0.057	0.666	Very weak
	EDSS	0.395	0.020*	Weak
	Anxiety score	0.714	< 0.001*	Strong
	Depression score	0.773	< 0.001*	Strong
**Quality of life index**	Age	-0.136	0.301	Very weak
	Body mass index	0.080	0.541	Very weak
	Duration of the disease	-0.007	0.959	Very weak
	EDSS	-0.587	< 0.001*	Moderate
	Anxiety score	-0.421	0.010*	Weak
	Depression score	-0.502	< 0.001*	Moderate
	Fatigue index	-0.551	< 0.001*	Moderate

Abbreviation: EDSS, Expanded Disability Status Scale.

Note: † Pearson correlation coefficient; *significant difference.

Regarding the treatments prescribed for multiple sclerosis, a comparison (depicted in Table 4 ) was undertaken between patients with and without fibromyalgia.

**Table 4:** Comparison of chosen treatments for multiple sclerosis among patients with and without fibromyalgia.

Characteristics	Fibromyalgia	Non-fibromyalgia	*p-value*
Current use of interferons	0	9	0.580
Previous/current use of interferons	0	27	0.013*
Current use of acetate	2	3	0.099
Previous/current use of acetate	2	14	1.000
Current use of oral medications	2	22	0.691
Previous/current use of oral medications	3	28	0.702
Current use of monoclonal antibodies	3	16	0.668
Previous/current use of monoclonal antibodies	3	17	0.676

Notes: Compared with the Fisher exact test (two-sided); *significant difference.

Qualitative variables are presented as absolute count.

## DISCUSSION

The present study aimed to assess the frequency of fibromyalgia in a series of adult patients with multiple sclerosis and the characteristics of such comorbidity, because of the lack of this kind of information in Brazil. As far as we know, our study is the pioneer on this theme outside Europe and North America. A total of 11.7% of our sample is affected by fibromyalgia, a prevalence relatively similar to those observed in other international case series from single centers in Italy (17.3% of 133 patients) and Turkey (19.4% of 103 patients). 5 6 An American survey covering a commercially insured population reported the combined outcome fibromyalgia/myalgia/myosistis as present in 12.5% of 5,000 patients with multiple sclerosis, a figure close to our result, although not defining precisely the actual frequency of fibromyalgia. 16

All aforementioned studies reported a higher prevalence than the survey performed in Manitoba, Canada, in which 6.8% of patients with multiple sclerosis also had the diagnosis of fibromyalgia, instead of the 3.5% found in the general population. 7 This was the only study that obtained a direct comparison of prevalence between multiple sclerosis patients and the general population, denoting the higher frequency of fibromyalgia in the formers. Although derived from different surveys in each country, it is possible that the estimate of the proportion of fibromyalgia among patients with multiple sclerosis is higher than that reported in the general population, namely: 2% in Brazil, 9 3.7% in Italy, 17 3.6% (female population) in Turkey, 18 and 5% in the United States. 19

Misdiagnosis is one of the concerns on the theme. In a series of 110 patients diagnosed with multiple sclerosis, 15% actually had fibromyalgia. 20 The high frequency of pain in multiple sclerosis may contribute to the misdiagnosis. In fact, the comorbidity of multiple sclerosis-associated pain and fibromyalgia was reported as 14% based on administrative claim records. 21 Such a fact emphasizes the importance of the appropriate recognition of each condition, as well as the awareness of how frequent the comorbidity is. Thermal and discomfort thresholds were lower in patients with multiple sclerosis than in controls and were the lowest in case of concomitant fibromyalgia. 22 It is possible that both conditions share central sensitization, but by different pathological mechanisms. 23 24

Two of the main findings or our study were the higher fatigue index and the lower quality of life index in patients with the comorbidity, compared with other patients only with multiple sclerosis. Fatigue is a common symptom of both multiple sclerosis and fibromyalgia, so it is not surprising that the association causes a higher fatigue index. Comorbidities, including fatigue, have a cumulative impact on quality of life in multiple sclerosis. 25 26

Depression and anxiety are the most common psychiatric conditions in multiple sclerosis, 27 28 occur more frequently than in the general population, 29 and are suggested as possible factors for enhancing disability. 30 Depression and anxiety scores were also higher in patients with the comorbidity in our study.

In order to explore the relations between these factors, we performed a correlation of quantitative variables with the fatigue index and the quality of life index. Anxiety and depression scores were strongly correlated to the fatigue index (the higher the scores, the worse the fatigue), but weakly and moderately to the quality of life index, respectively, in an inverse manner (the higher the scores, the worse the quality of life). As expected by the literature, 25 there was an inverse correlation between the fatigue and the quality of life indices in our sample.

The EDSS is widely used to measure disability in demyelinating diseases of the CNS. The difference in the EDSS score between the comorbidity group and other patients with multiple sclerosis just lost significance in the statistical analysis, but this may be a limitation of our sample size. A previous survey found higher EDSS scores in the comorbidity group compared with patients with multiple sclerosis without any pain, but no significant difference among these groups with patients with multiple sclerosis who suffered from non-fibromyalgic chronic pain. 22 We found a weak correlation of the EDSS score with the fatigue index, whilst the correlation was moderate and inverse with the quality of life index. All these correlations do not prove causation but indicate that there is some relation between the variables.

Another interesting finding has emerged from the analysis: those who were diagnosed with fibromyalgia have never been prescribed interferon, despite the diagnosis of the pain syndrome having been established only later. We interpreted that the neurologists who assisted these patients with multiple sclerosis probably considered the complaint of pain as a factor for avoiding the prescription of interferons, considering the known adverse effects of these medications, including pain.

The results above emphasize the importance of recognizing fibromyalgia among patients with multiple sclerosis. Fatigue, quality of life, depression, and anxiety may be worse in the presence of the comorbidity and the symptoms of recognized or unrecognized fibromyalgia may influence the choice of treatment for multiple sclerosis. In our opinion, this is enough to recommend an active search for the diagnosis of the pain syndrome also by the neuroimmune practitioner.

The main limitation or our study is the sample size, as aforementioned. There is also lack of information from the pediatric population, but this is an issue shared with previous reports, because no study evaluated the comorbidity in children and adolescents.

In conclusion, the present survey pointed to the existence of an important comorbidity of fibromyalgia and multiple sclerosis also in Brazil, and brought some information regarding distinctive clinical characteristics of patients with both conditions and the pertinence of recognizing this pain syndrome for a more adequate management of these patients.
